# Current Status of Anisakiasis and *Anisakis* Larvae in Tokyo, Japan

**DOI:** 10.14252/foodsafetyfscj.D-21-00004

**Published:** 2021-12-07

**Authors:** Jun Suzuki, Rie Murata, Yukihiro Kodo

**Affiliations:** Department of Microbiology, Tokyo Metropolitan Institute of Public Health, 3-24-1 Hyakunin-cho, Shinjuku-ku, Tokyo 169-0073, Japan.

**Keywords:** food poisoning, parasitological survey, *Anisakis simplex* sensu stricto, *Anisakis pegreffii*, *Katsuwonus pelamis*, food safety

## Abstract

Anisakiasis is a gastrointestinal disease caused by infection with anisakid nematodes. *Anisakis* larvae have been listed as distinct food poisoning agents in the manual of Food Poisoning Statistics, Japan since 2013. The reported numbers of food poisoning cases caused by *Anisakis* larvae are gradually increasing. A total of 94.0% of the causative larvae species were identified as *Anisakis simplex* sensu stricto (*A. simplex*), and 4.4% were identified as *Anisakis pegreffii*, among human-isolated anisakid nematodes examined in Tokyo Metropolitan Institute of Public Health, Japan from 2011 to 2018. *Anisakis* species infecting fishes in Japanese waters differ depending on their habitat and depth. *A. simplex* mainly infects fishes in the Pacific side of Japan, and *A. pegreffii* mainly infects fishes in the East China Sea and Sea of Japan sides. Regarding the causative foods of anisakiasis, cases by ingestion of mackerel (*Scomber* spp.) have been the most common in Japan, and cases caused by eating “marinated mackerel” accounted for 32.8% of the total in Tokyo from 2011 to 2017. However, the number of reports of food poisoning caused by skipjack tuna (*Katsuwonus pelamis*) was highest in May 2018 in Japan. A parasitological surveys of *Anisakis* third-stage larvae in skipjack tuna in Japanese waters were conducted in 2018 and 2019, and it was confirmed that more *A. simplex* infections of skipjack tuna may have occurred in 2018 than usual due to the meandering flow of the Black Current. Moreover, a portion of *A. simplex* larvae migrated from visceral organs to the ventral muscle in live skipjack tuna before capture, suggesting that an extensive cold chain after capture cannot prevent anisakiasis. In fish species that were reported to be high frequency of causative food of anisakiasis, it is necessary to freeze or at least remove the ventral muscle.

## Introduction

Anisakid nematodes have been included as pathogenic agents in the Food Sanitation Law of Japan since 1999. Subsequently, in 2013, *Anisakis* larvae were listed as distinct food poisoning agents in the Manual of Food Poisoning Statistics, Japan, requiring medical doctors to report cases to a public health center. As a result, among the food poisoning incidents that have increased annually (Table 1), we have become to more clearly understand about symptoms, causative fishes and origins associated with food poisoning caused by *Anisakis* larvae (*Anisakis* food poisoning). Furthermore, anisakiasis is the most common form of food poisoning since 2017, having its case numbers exceeded *Campylobacter jejuni/coli* and *Norovirus* infections. These three food poisonings occupied 76.1% of the total case numbers in duration of 2015 through 2019. However, unlike many other food poisonings, 97.6% (1417/1452 cases) of food posoning anisakiasis cases were single patient cases. Moreover, the food poisoning cases caused by *Anisakis* spp. constitute only a part of total cases of anisakiasis, and the number of anisakiasis cases based on data from medical institutions is estimated to be 7,000 cases per year in Japan^1^^)^. In this review, we will go over anisakiasis in Japan, mainly in Tokyo, and parasitological surveys of *Anisakis* third-stage (*Anisakis* L3) larvae in fish, especially skipjack tuna (*Katsuwonus pelamis*) and Pacific bluefin tuna (*Thunnus orientalis*) (PBT).

**Table 1.** Number of *Anisakis* food poisoning cases and patients in Japan from 2013 to 2020 based on food poisoning statistics from the MHLW

Year	No. of cases	No. of patients
2013	88	89
2014	79	79
2015	127	133
2016	125	127
2017	234	246
2018	469	479
2019	330	338
2020	387	397
Total	1839	1888

## 1. *Anisakis* third-stage Larvae in Fish

Nematodes of the genus *Anisakis* are parasites, whose definitive hosts are marine mammals such as whales and dolphins, marine fish, and squid. They serve as paratenic hosts for *Anisakis* L3 larvae. Nine species of *Anisakis* nematodes are known^2^^,^^3^^,^^4^^,^^5^^,^^6^^,^^7^^,^^8^^,^^9^^)^, and *Anisakis* L3 larvae are divided into Type I–IV based on their morphological differences^10^^)^. The larvae of six species, namely, *Anisakis simplex* sensu stricto (*A. simplex*), *Anisakis pegreffii (A. pegreffii)*, *Anisakis berlandi*, *Anisakis typica (A. typica)*, *Anisakis ziphidarum* and *Anisakis nascettii*, are classified as Type I larvae because of their morphological similarity. It has been reported that *A. pegreffii* has a slightly shorter ventriculus length than *A. simplex*^11^^)^. On the other hand, *Anisakis* Type II, Type III and Type IV larvae may be morphologically identified as *Anisakis physeteris (A. physeteris)*, *Anisakis brevispiculata* and *Anisakis paggiae*, respectively^12^^)^. Eight out of the nine *Anisakis* species, after excluding *A. nascettii*, can be detected in fish caught in Japanese waters, as has been confirmed in our survey. There is no report of *A. nascettii* being detected in Japan.

**Fig. 1.**
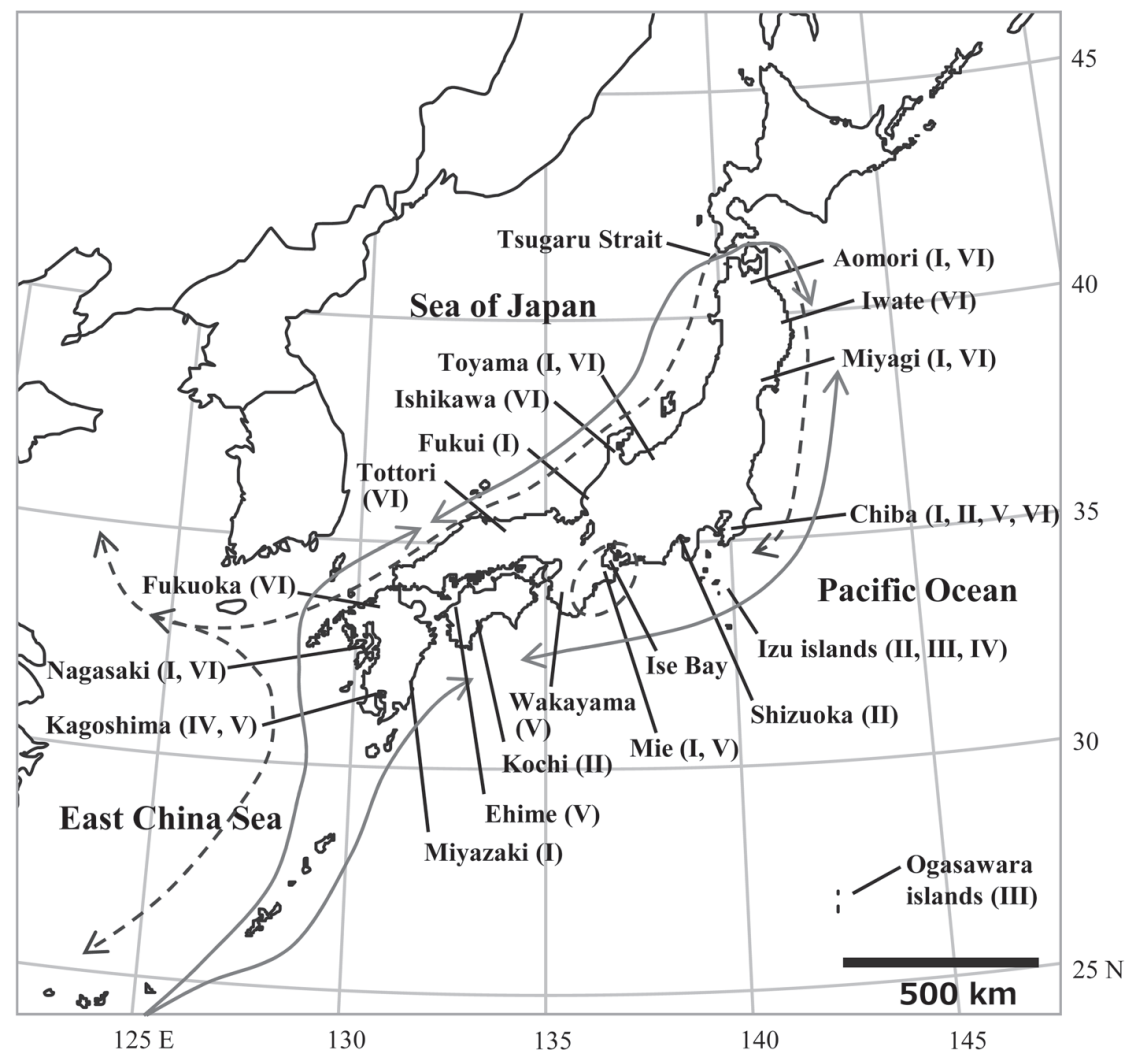
Localities of landing fishes in Japanese waters I: Districts of Japanese Spanish mackerel *(Scomberomorus niphonius*), II: Districts of splendid alfonsino (*Beryx splendens*), III: District of deep-water red snapper *(Etelis carbunculus*), IV: District of deep-water longtail red snapper (*Etelis coruscans*), V: Districts of hairtail (*Trichiurus lepturus*), VI: Districts of juvenile PBT (*Thunnus orientalis*). Dotted arrow line indicate Japanese Spanish mackerel migration routes^16^^)^, while arrowed lines indicate juvenile PBT migration routes^45^^,^^46^^,^^47^^,^^48^^)^.

*Anisakis* species infecting fishes in Japanese waters differ depending on their habitat and depth. According to a survey of chub mackerel (*Scomber japonicas*), *A. simplex* mainly infects chub mackerel in the Pacific side of Japan, while *A. pegreffii* mainly infects chub mackerel in the East China Sea and the Sea of Japan sides^13^^)^. This trend is often found in not only chub mackerel but also other fish species^14^^,^^15^^)^. Fishing area and habitat depth as well as migration route affect on the *Anisakis* species in migratory fish. For example, in Japanese Spanish mackerel (*Scomberomorus niphonius*) caught in the Pacific Ocean side of Japan (details described in supplemental materials and methods), *A. pegreffii* was the main species detected (Table 2). This is because, although the fishes were caught on the Pacific side, they were originally born in the East China Sea and passed through the Tsugaru Strait from the Sea of Japan side (Fig. 1; dotted arrow line)^16^^)^. *A. simplex* was the main species infecting Japanese Spanish mackerel shipped from Mie prefecture. They were thought to be born and grown around Ise Bay^17^^)^ (Table 2). Among deep-sea fishes such as *Beryx splendens*, *Etelis carbunculus* and *Etelis coruscans*, *A. physeteris* was frequently detected (Table 3; methods for *E. carbunculus* and *E. coruscans* were described in supplemental materials and methods). *A. typica* was detected at a high rate in hairtail (*Trichiurus lepturus*) that were landed at sea ports of Kagoshima prefecture (Table 4; details described in supplemental materials and methods), and similarly reported as mainly collected from hairtail of Taiwan captures^18^^)^. In most of the fish species in which *Anisakis* larvae were detected in the muscle, the muscular part in question was the ventral muscles. Detection of the larvae in the dorsal muscle was limited to species such as mackerel and natural salmon (*Oncorhynchus keta*), and almost all the larvae detected in the muscle were identified as *A. simplex*.

**Table 2.** Number of *Anisakis* larvae detected in Japanese Spanish mackerel (*Scomberomorus niphonius*) from Japanese waters from 2007 to 2009

Catching area	District of fishby prefecture	No. of fish samples (positive)	No. of *Anisakis* larvae		
			*A. simplex *sensu stricto	*A. physeteris*	Total
Pacific Ocean	Aomori	2 (1)	0	1	1
	Miyagi	1 (1)	0	1	1
	Chiba	10 (6)	0	19	19
	Mie	3 (1)	14	0	14
Sea of Japan and East China Sea	Nagasaki	2 (2)	0	8	8
	Miyazaki	2 (2)	0	3	3
	Fukui	2 (1)	0	4	4
	Toyama	12 (9)	1	82	83
Total		34 (23)	15	118	133

**Table 3.** *Anisakis* species detected in deep-sea fishes from Japanese waters

Fish species (examination periods)	District of fish	Fish samples (positive)	No. of *Anisakis* larvae (%)						Total larvae
			*A. simplex *sensu stricto	*A. berlandi*	*A. ziphidarum*	*A. physeteris*	*A. brevispiculata*	*A. paggiae*	
*Beryx splendens*^12^^)^ (2007-2009)	Tokyo (Izu islands)	8 (8)	11	1	0	107	2	2	123
	Chiba	9 (9)	4	0	0	31	1	1	37
	Shizuoka	19 (19)	21	4	1	403	6	44	479
	Kochi	8 (8)	6	0	0	83	1	1	91
*Etelis carbunculus* (2012-2014)	Tokyo (Izu-Ogasawara islands)	12 (9)	2	0	0	58	0	0	60
*Etelis coruscans* (2012-2014)	Tokyo (Izu islands)	17 (7)	2	0	1	14	0	0	17
	Kagoshima	2 (1)	0	0	0	3	0	0	3
Total		75 (62)	46 (5.7)	5 (0.6)	2 (0.2)	699 (86.3)	10 (1.2)	48 (5.9)	810 (100)

**Table 4.** Detection numbers of *Anisakis* larvae in hairtail (*Trichiurus lepturus*) from Japanese waters from 2013 to 2015

District of fish	No. of fish samples (positive)	No. of *Anisakis* larvae			Total larvae
		*A. simplex*sensu stricto	*A. typica*	*A. physeteris*	
Chiba	3 (3)	13	0	0	13
Mie	5 (0)	0	0	0	0
Wakayama	3 (0)	0	0	0	0
Ehime	6 (0)	0	0	0	0
Kagoshima	7 (3)	0	47	7	54
Total	24 (6)	13	47	7	67

## 2. Anisakiasis and Food Poisoning Caused by *Anisakis* larvae in Tokyo, Japan

Consumption of seafood prepared using raw fish and raw fish-alike (such as sashimi, sushi and marinated fish) containing *Anisakis* L3 larvae may let them invade into the stomach and intestinal walls to cause acute gastroenteritis in humans. This gastroenteritis is known as anisakiasis. Anisakiasis was for the first time reported in the Netherlands^19^^) ^in 1960, and in Japan^20^^)^ in 1965. Anisakiasis, as a result of *Anisakis* food poisoning, more frequently occur in households than most of other types of food poisonings based on food poisoning statistics in Japan. This tendency became clearer in year 2020 food poisoning statistics, possibly attributing to the requests issued by the Japanese government and local governments to refrain from eating out during the COVID-19 outbreak in Japan and people’s compliance during year 2020. In 2020, the number of food poisoning cases caused by *Campylobacter jejuni/coli* and *Norovirus* decreased by 43% and 58% year-on-year, respectively, but the number of *Anisakis* food poisoning cases increased by 17.3% from the previous year.

Anisakiasis clinical cases are divided into fulminant and mild forms according to their severity of symptoms. Mild form is often asymptomatic and rarely diagnosed. The fulminant form is further divided into two main types of gastric anisakiasis and intestinal anisakiasis, depending on the locations of *Anisakis* larvae. Moreover, there are also a few reports of gastro-allergic anisakiasis associated with *A. simplex* and *A. pegreffii* larvae^21^^,^^22^^)^. Onset of clinical symptoms in acute gastric anisakiasis cases was reported to occur within 1 to 12 h after the ingestion of infected fish, with peaking at 6 h^23^^)^. In accordance, onset of anisakiasis cases examined at the Tokyo Metropolitan Institute of Public Health from 2011 to 2017 showed, among 180 cases (179 gastric anisakiasis cases and an intestinal anisakiasis case), 91.2% (134/147 cases) with clearly known onset timing from the ingestion occurred within 12 h (Table 5). The main symptoms such as abdominal pain (100%), nausea (68.3%), vomiting (39.0%), diarrhea (19.5%), and urticarial (9.8%) were reported^24^^)^.

**Table 5.** Time to onset of clinical symptoms in anisakiasis cases at Tokyo Metropolitan Institute of Public Health from 2011 to 2017

Time	No. of cases (%)
Less than 4 h	45 (25.0)
4 to 8 h	59 (32.8)
8 to 12 h	30 (16.7)
More than 12 h*	13 (7.2)
Unknown**	33 (18.3)
Total	180 (100)

*Maximum time was 39 h. **Cases where raw fish was eaten multiple times within 2 days or unanswered cases.

**Fig. 2.**
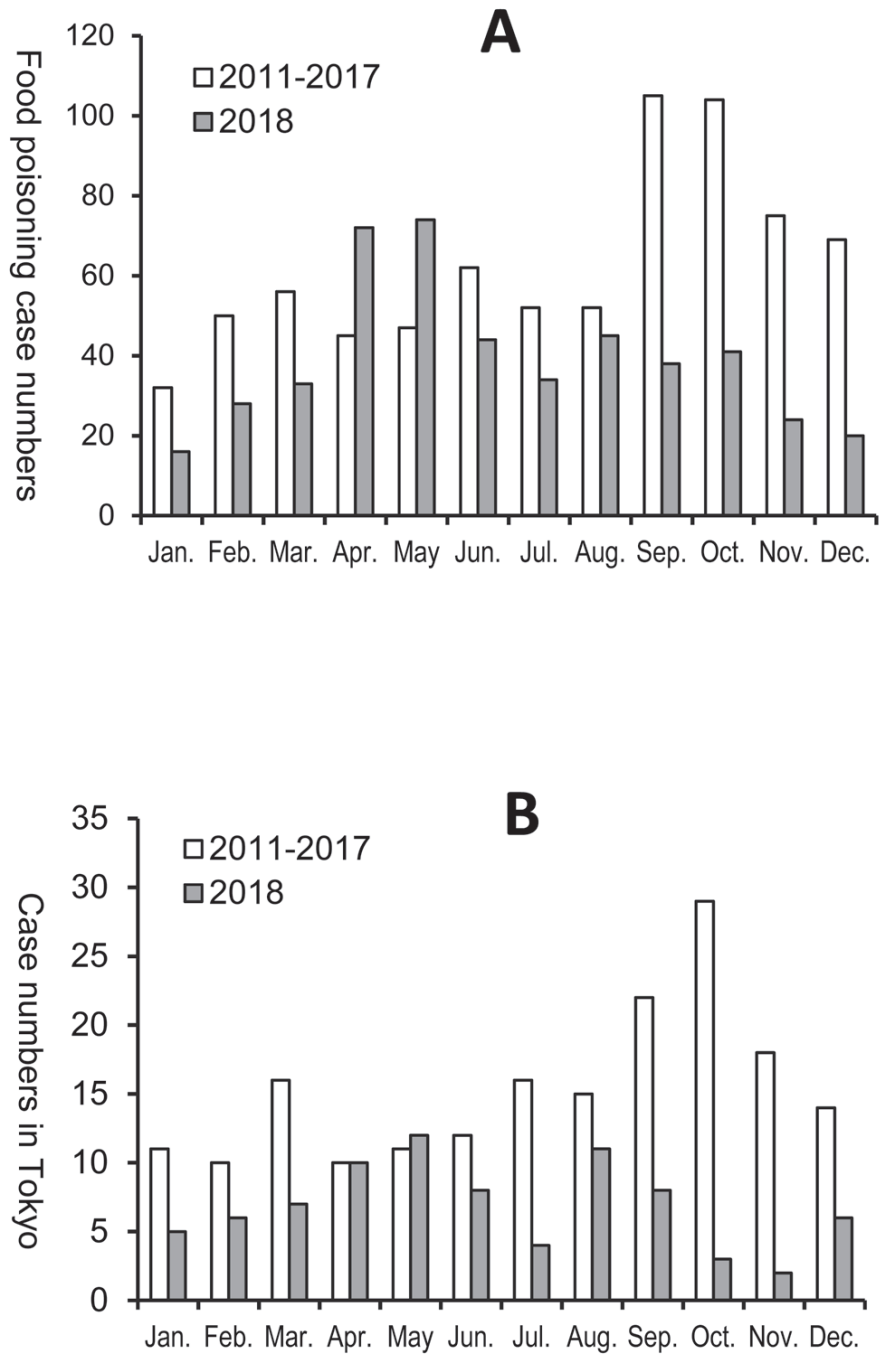
Food poisoning cases caused by *Anisakis* spp. in Japan (A) and anisakiasis cases at the Tokyo Metropolitan Institute of Public Health (B). A: Graph was created based on numbers of *Anisakis* food poisoning cases in the manual of Food Poisoning Statistics, Japan. (https://www.mhlw.go.jp/stf/seisakunitsuite/bunya/kenkou_iryou/shokuhin/syokuchu/04.html), B: Anisakiasis case numbers examined at the Tokyo Metropolitan Institute of Public Health^25^^)^.

According to an interview-based survey conducted by public health centers’ staff in Tokyo from 2011 to 2017, mackerel was the most important causative food and accounted for 42.8% of anisakiasis cases’ reasons in Tokyo, followed by marinated mackerel accounting for 32.8%^25^^)^. In the monthly statistics^25^^)^ that were reported to Ministry of Health, Labour and Welfare of Japan (MHLW) regarding *Anisakis* food poisonings from 2011 to 2017, the case peak took place in September and October (Fig. 2), because of seasonally consumption saury (*Cololabis saira*) as a cause anisakiasis. It has been reported that *A. simplex* was detected in 13% of saury’s visceral organs and 1.8% of their muscle tissues^26^^)^. This detection rate was lower than those in mackerel^13^^)^ and natural salmon^27^^)^, however, considering the number of *Anisakis* food poisoning cases by ingestion of saury, the detection rate in the muscle should not be ignored. In 2018, the number of *Anisakis* food poisoning cases in Japan peaked in April and May (Fig. 2), unlike in the regular trend. Moreover, in many of these cases, skipjack tuna was presumed to be the causative fish of *Anisakis* food poisoning. In cases where *Anisakis* spp. were identified at the Tokyo Metropolitan Institute of Public Health, 23 anisakiasis cases had a history of skipjack tuna ingestion in 2018, among which 13 cases were with a sole consumption of skipjack tuna^25)^. This was only second to 33 cases confirmed to have ingested mackerel, among which 13 cases were with a sole consumption of mackerel (Table 6, numbers of food poisoning cases of mackerel; unpublished data). 

**Table 6.** Numbers of anisakiasis cases with a history of skipjack tuna (*Katsuwonus pelamis*) and mackerel (*Scomber* spp.) ingestion in 2018, and the numbers of larvae detected per patient in Tokyo, Japan

No. of *Anisakis* larvae per patient	Skipjack tuna (ingestion of only skipjack tuna)	Mackerel* (ingestion of only mackerel)
*A. simplex* sensu stricto		
1	19 (11)	24 (8)
2	2 (1)	7 (4)
3	1 (1)	0
4	0	1 (0)
5	1 (0)	0
*A. pegreffii*		
1	0	1 (1)
2	0	0
Total cases	23 (13)	33 (13)

Anisakiasis cases were examined numbers at the Tokyo Metropolitan Institute of Public Health.

*Mackerels included in *Scomber japonicus* and *S. australasicus*.

## 3. Causative *Anisakis* species in Anisakiasis

Out of 318 anisakid nematodes from patients examined at the Tokyo Metropolitan Institute of Public Health from 2011 to 2018 period^28)^, 299 larvae (94.0%) were identified as *A. simplex* and 14 larvae (4.4%) as *A. pegreffii* (Table 7). It has been reported that the causative *Anisakis* species of anisakiasis is *A. simplex,* even in Kyushu region, where the detection rate of *A. pegreffii* such as in mackerel is higher than that in Pacific coast regions^29^^)^. The reason for this epidemiological trend was the migration rate of *A. simplex* from the visceral organs to the muscle of chub mackerel being 100 times faster than that of A. pegreffii^13)^ shown in Table 8. A comparison of penetration abilities by *A. simplex* and *A. pegreffii* using agar confirmed that *A. simplex* had higher rate of agar penetration than *A. pegreffii*^13^^,^^30^^)^. Moreover, a study with experimental infection in Wistar rats reported that the tissue penetration rate of *A. simplex* was 63% higher than that of *A. pegreffii*^31^^)^. However, the clinical symptoms of fulminant anisakiasis caused by *A. simplex* and *A. pegreffii* are similar, characterized by epigastric pain, nausea, vomiting and abdominal fullness, and there is no significant difference in histopathological findings between the two species. Therefore, *A. simplex* is not greater pathogenic than *A. pegreffii* but migration capability in the fish muscle is greater. Because of increased opportunities to consume *A. simplex*, it is a major causative agent of anisakiasis. Anisakiasis caused by *Anisakis* species other than *A. simplex* and *A. pegreffii* in Japan was reported; in one case, *A. typica* was identified as a cause (The 86^th^ Annual Meeting of the Japanese Society of Parasitology, 2017), and in two cases, *A. physeteris* was identified as causes^32^^,^^33^^)^.

**Table 7.** Number of human isolate anisakid nematodes identified in Tokyo Metropolitan Institute of Public Health from 2011 to 2018

Larval species	2011	2012	2013	2014	2015	2016	2017	2018	Total (%)
*A. simplex* sensu stricto	11	30	30	24	23	27	56	98	299 (94.0)
*A. pegreffii*	0	0	0	0	2	2	3	7	14 (4.4)
*Pseudoterranova azarasi*	0	1*	0	1*	0	0	2	1	5 (1.6)
Total larvae	11	31	30	25	25	29	61	106	318 (100)

*Pseudoterranova sp. (genetic analysis not performed)

**Table 8.** Number of *Anisakis* larvae detected in chub mackerel (*Scomber japonicus*) from Japanese waters^13^^)^

Catching area	Main *Anisakis* species	No. of fish samples (positive)	Total no. of *Anisakis* larvae (A)	No. of larvae in fish muscle (B)	Percentage of larvae in fish muscle (B/A)
Sea of Japan and East China Sea	*A. pegreffii*	86 (63)	4073	4	0.1% (4/4073)
Pacific Ocean	*A. simplex* sensu stricto	132 (99)	733	81	11.1% (81/733)

**Fig. 3.**
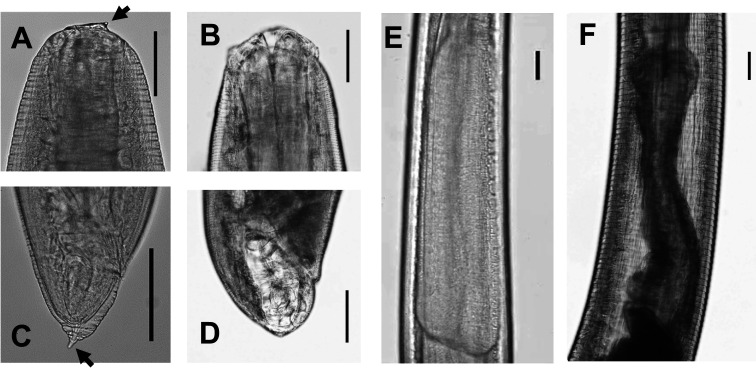
Morphology of third-stage (L3) and fourth-stage (L4) larvae of *Anisakis simplex* sensu stricto A, Cephalic end of L3 larva (arrowed line: boring tooth); B, Cephalic end of L4 larva; C, Caudal end of L3 larva (arrowed line: mucron); D, Caudal end of L4 larva; E, Ventricular part of L3; F, Ventricular part of L4. Bar: 100 μm.

Among 208 *Anisakis* larvae detected in patients who were examined in our institute from 2011 to 2017, seven larvae were morphologically fourth-stage *Anisakis* (*Anisakis* L4) larvae, and 59 larvae (28.4%) were difficult to identify for their stages based because of damages for morphological examination. Morphological characteristics between *Anisakis* L3 and L4 in Type I larvae are very different^34^^,^^35^^)^. Both the boring tooth at the anterior end and mucron at the posterior tip in morphological Type I larvae in the *Anisakis* L3 (Fig. 3-A and C) are absent in the *Anisakis* L4, and perioral lips are present at the anterior of L4 larvae (Fig. 3-B and D). L3 larvae possess a relatively long ventriculus with an oblique ventricular-intestinal junction, but the ventriculus of L4 larvae shows morphological changes in the internal structure^36^^).^Fig. 3-E and F: Table 1**and**Fig. 3 display morphological L3 larvae. The maximum number of larvae detected in one patient was 10, and the suspected causative food was natural salmon. In 91.7% of cases (165/180), the number of larvae removed from per patient was one. The reporting of *Anisakis* food poisoning by each local government to the MHLW is often based on morphological examination of larvae, and a few local governments carry out molecular identification. Since the *Anisakis* species infecting fishes are closely associated with the catch area and habitat of the fishes, it is important to identify the larval species, and narrow down or determine the causative fish. It necessitates active conduct of molecular identification of larvae detected in humans.

## 4. Survey of *Anisakis* larvae from Skipjack Tuna, *Katsuwonus pelamis*

To investigate the cause of increase in *Anisakis* food poisoning cases by ingestion of skipjack tuna in 2018, a parasitological survey of *Anisakis* L3 larvae was conducted in skipjack tuna^25^^)^ from May 2018 to October 2019.

In 2018, the survey investigated the timing of *Anisakis* migrates from visceral organs to the muscle of skipjack tuna, whether it took place before catching or after, a parasitological examination was conducted regarding *Anisakis* larvae between August and November by comparison among different timings of gutting before processing for sale. *Anisakis* larvae were detected not only in the muscle after landing, but also found in the muscle of skipjack tuna, in which the visceral organs were immediately removed after catching^25^^)^. *Anisakis* larvae, when found in the muscle, were only in the ventral muscle. All the larvae in the ventral muscle were identified as *A. simplex*. These findings demonstrated, with a portion of *A. simplex* larvae migrated to the muscle in live skipjack tuna before capture, that an extensive cold chain after capture does not prevent anisakiasis.

Out of 10 skipjack tunas caught off the east coast of Chiba prefecture in May 2018, 13 *Anisakis* L3 larvae were detected in the ventral muscle of 2 fishes. In a total of 680 *Anisakis* L3 larvae were collected from 88 out of 90 skipjack tunas from August to November 2018 period^25)^, where, 47 larvae were detected in the muscle of 14 fishes (Table 9). Out of 21 skipjack tunas caught off the coasts of Mie and Shizuoka prefectures between April and June 2019, a total of 115 *Anisakis* L3 larvae were detected in 19 fishes, and 2 larvae were detected in the ventral muscle of two fishes. Out of 30 skipjack tunas caught off the coasts of Chiba and Miyagi prefectures between July and October 2019, 494 *Anisakis* L3 larvae were detected from 29 fishes and 8 larvae from the ventral muscle of six fishes^25)^ as in Table 9. In the present survey from May 2018 to October 2019, the most common *Anisakis* species was *A. simplex* (1016/1421 larvae), and all the larvae detected in the muscle were identified as *A. simplex*. The second most frequently detected species was *A. physeteris* in this survey. It was recently reported that skipjack tuna prey on fish even at a depth of 200 meters^37^^)^, explaining why *A. physeteris* was detected in skipjack tuna.

**Table 9.** Number of *Anisakis* larvae detected in skipjack tuna (*Katsuwonus pelamis*) from May 2018 to October 2019^25^^)^

Period	Fish samples (A)	Positive samples (in muscle)	No. of *Anisakis* larvae		%, larvae in fish muscle (B/C)	Mean abundance of larvae in fish muscle (B/A)
			In muscle* (B)	Total (C)		
May, 2018	10	8 (2)	13	132	9.80%	1.3
					(13/132)	(13/10)
Aug to Nov, 2018	90	88 (14)	47	680	6.90%	0.52
					(47/680)	(47/90)
Apr to Jun, 2019	21	19 (2)	2	115	1.70%	0.1
					(2/115)	(2/21)
Jul to Oct, 2019	30	29 (6)	8	494	1.60%	0.27
					(8/494)	(8/30)

*All larvae in fish muscle were *Ansiakis simplex* sensu stricto.

Current status of anisakiasis and *Anisakis* larvae in Tokyo, Japan (Jun Suzuki, Rie Murata, Yukihiro Kodo)

In May 2018, the mean abundance and percentage of *A. simplex* larvae in the ventral muscle of skipjack tuna were 1.30 (13 larvae/10 fishes) and 9.8% (13 larvae/132 larvae), respectively^25)^, and these values were higher than those in the other study periods (Table 9). Because the skipjack tuna was obtained from a single locality (off Chiba) in May 2018 and was caught in an area of the ocean approximately 500 km away from the main area of fish catch in April and May 2018, when *Anisakis* food poisoning cases caused by the ingestion of skipjack tuna were reported, there was insufficient evidence supporting that the skipjack tuna examined in April and May 2018 were infected with *A. simplex*. However, because the number of *A. simplex* larvae in the ventral muscle of skipjack tuna in 2018 was higher than that in 2019, it was considered that *Anisakis* food poisoning caused by ingestion of skipjack tuna was reported more frequently than usual in 2018.

The difference in the number of *Anisakis* larvae in “Hatsugatsuo” (the season’s first skipjack tuna in April and June) between 2018 and 2019 may be related to the host migratory patterns. As skipjack tuna inhabit sea waters at temperatures of >18°C, those around Japan may migrate north via three major routes as the ocean water temperature increases: along with the Black Current, around the Izu-Ogasawara Islands, and more extensively, in the Pacific Ocean on the east side of the Izu Islands^38^^,^^39^^,^^40^^)^. The fishing area of “Hatsugatsuo” is usually the sea around the Ogasawara Islands in April and around the Izu Islands in May. In 2018, the Black Current took a large meandering path along the Pacific coasts and increased the temperature of the sea around Miyakejima Island^41^^)^, the main fishing area of skipjack tuna in April 2018, to above 18°C, which is relative to the sea temperature (18°C) in May 2017. Some schools of skipjack tuna may have migrated north earlier than usual in 2018. This may have changed their feeding habits, causing an increase in *Anisakis* infection. *Anisakis* food poisoning was reported in Tokyo, Fukushima and Miyazaki prefectures, as well as elsewhere, at the same time, which was thought to be a result of skipjack tuna being caught in the same sea area and distributed across Japan.

As part of an investigation into the cause of the increase in anisakiasis by the ingestion of skipjack tuna in 2018, a questionnaire survey of 102 fish and shellfish distributors and 339 restaurant businesses in Tokyo was conducted by the public health center staff in Tokyo between October and December 2018, where they were asked whether skipjack tuna had been frozen^25^^,^^42^^)^. Among the fish and shellfish distributors (102 facilities), 47 (46.1%) and 35 (34.3%) facilities distributed only either fresh chilled or frozen skipjack tuna, respectively, whereas 20 (19.6%) distributed both. Of restaurant businesses (339 facilities), 254 (74.9%) and 30 (8.8%) facilities served only either fresh chilled or frozen skipjack tuna, respectively, whereas 30 (9.0%) served both. Moreover, of 67 fish and shellfish distributors trading in chilled raw or semi-raw skipjack tuna, the survey revealed that 45 of the 67 facilities (67.2%) removed the ventral muscle. On the other hand, among 284 restaurant businesses that served chilled raw or semi-raw skipjack tuna, 165 facilities (58.1%) removed the ventral muscle. From the questionnaire survey, it was revealed that fish and shellfish distributors had paid more attention to prevention of *Anisakis* food poisoning by skipjack tuna than restaurant businesses.

## 5. *Anisakis* larvae in Juvenile PBT, *Thunnus orientalis*

It is known that *Thunnus* spp. and skipjack tuna (*Katsuwonus pelamis*) are migratory fish and belong to the same clade in the morphological phylogeny of the family Scombridae^43^^)^. PBT (*Thunnus orientalis*) is called “Honmaguro” in Japan, and the muscle of adult PBT is an expensive seafood used for sushi and sashimi. On the other hand, juvenile PBT are often accidentally caught in Japanese waters and can generally be purchased in markets at lower prices. Ingestion of tuna has sometimes been reported as a cause of anisakiasis in interview-based surveys conducted by public health center staff in Tokyo, because tuna is included in many sashimi platters and not only in single dishes such as tuna bowls and sushi. However, there are few reports in Japan of *Anisakis* larvae in PBT, and we had only two cases thus far, in which *Anisakis* larvae were detected from patients or tuna leftovers.

**Fig. 4.**
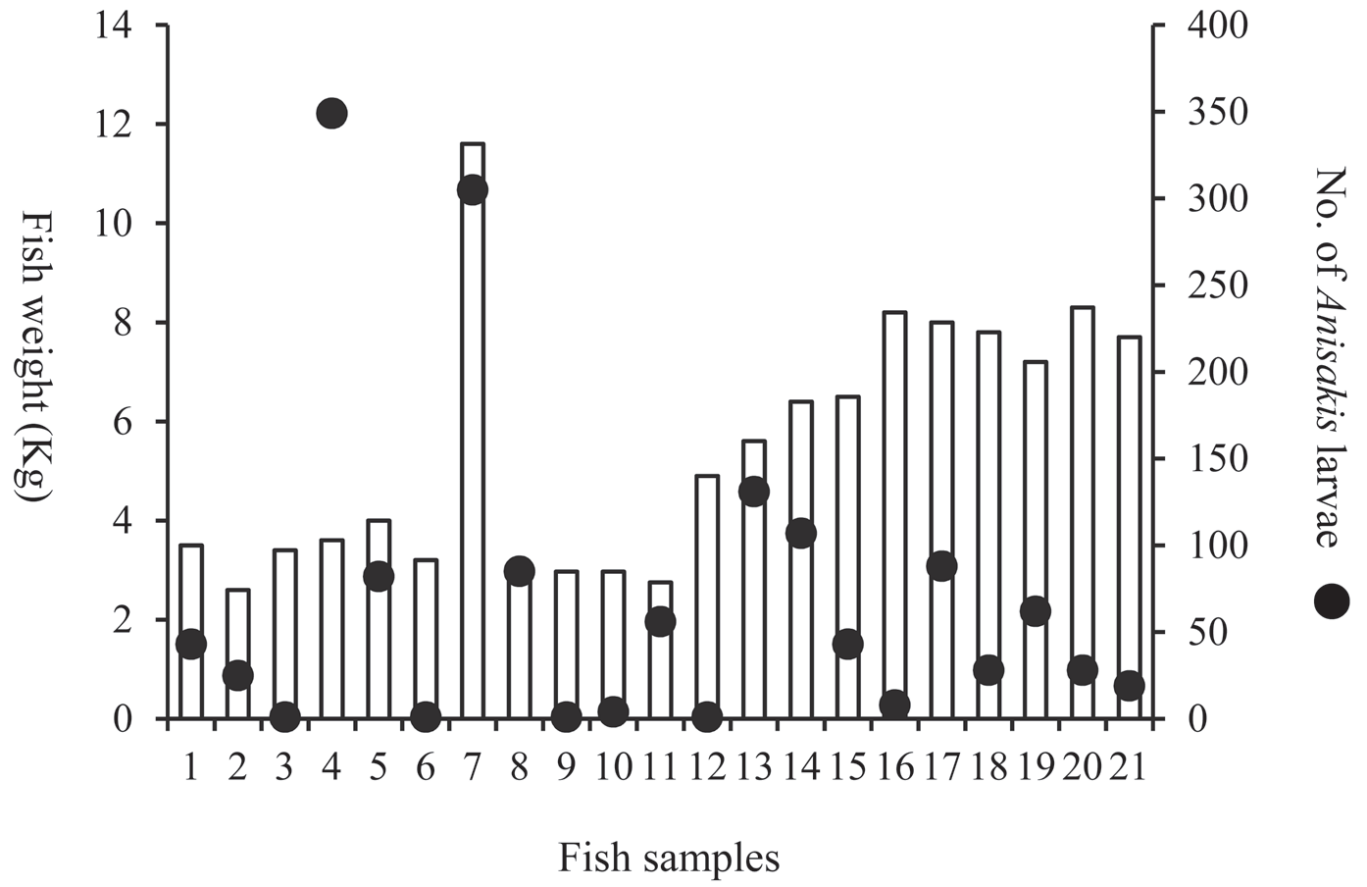
Association between the number of *Anisakis* larvae and the weight of juvenile PBT in Japanese waters from 2005 to 2006^44^^)^

A parasitological survey of *Anisakis* larvae from 39 juvenile PBT (1.4 kg to 11.6 kg) was conducted in 2005 to 2006, and 1,467 *Anisakis* larvae detected in 21 fishes were genetically identified. No correlation was found between the weight of juvenile PBT and the number of larvae in this study^44)^ (Pearson’s correlation test, *P* > 0.05) (Fig. 4). Moreover, a parasitological survey of *Anisakis* larvae from 104 juvenile PBT (1.5 kg to 12.7 kg) was conducted from 2011 to 2013 (Fig. 1), and *Anisakis* larvae detected in 30 fishes were genetically identified. In this parasitological survey, a total of 302 larvae were detected in 16 juvenile PBT caught in the Sea of Japan and the East China Sea, of which 295 larvae (97.7%) were identified as *A. pegreffii*. A total of 143 larvae were detected in 14 juvenile PBT caught in the Pacific Ocean side, of which 130 larvae (90.9%) were also identified as *A. pegreffii,* and there was no difference in *Anisakis* species depending on the catch areas (manuscripts in preparation). PBT spawn around the Nansei Islands, grow in the Sea of Japan side (off Shimane prefecture to off Toyama prefecture) while migrating around Japanese waters (Fig. 1), and eventually cross the Pacific Ocean and reach the west coast of North America^45^^,^^46^^,^^47^^,^^48^^)^. PBT larvae are reported to prey primarily on plankton such as copepods^49^^)^. As PBT grows, it begins to prey on small fish and grows rapidly^50^^,^^51^^)^. The number of *Anisakis* larvae in juvenile PBT was expected to increase as a fish grew; however, no correlation was found between the weight of juvenile PBT and the number of larvae in this study. Since 90.9% of the *Anisakis* larvae detected in juvenile PBT caught on the Pacific side of Japan were identified as *A. pegreffii* and the number of larvae did not increase with fish weight, it was suggested that the juvenile PBT may have been infected with *Anisakis* spp. larvae during the fish larval period. The risk of anisakiasis due to ingestion of juvenile PBT is considered to be lower than that of skipjack tuna because more than 90% of the *Anisakis* larvae detected in juvenile PBT were *A. pegreffii* and no larvae were detected in the fish muscle.

## 6. Concluding Remarks

It is recommended by the MHLW that *Anisakis* larvae are killed by freezing in fish at < –20°C for 24 h or heating at > 60°C for 1 minute. The Food and Drug Administration (2020) recommends freezing fish at −20°C or below for 7 days or −35°C or below until solid, then storing at −35°C for 15 h, −35°C or below until solid, or at −20°C or below for 24 h or by cooking adequately to an internal temperature risen at least to > 63°C^52^^)^. Appropriate freezing and cooking are the most reliable measures to prevent *Anisakis* food poisoning; however, there are some fish species whose commercial values are significantly reduced by such measures. Therefore, it will be necessary to monitor not only *A. simplex* infection but also the larvae migrating in fish muscle, which is frequently reported as a source of infection. We recommend removal of the ventral muscle to prevent anisakiasis according to the fish species and the fishing area when a high frequency of *A. simplex* is detected in fish. Furthermore, it is necessary to pay close attention to the trends of anisakiasis and the causative foods, and to alert not only fish and shellfish distributors and restaurant businesses but also the general public regarding measures to prevent anisakiasis.
